# Dental Materia Medica and Therapeutics

**Published:** 1910-02-15

**Authors:** 


### BIBLIOGRAPHY.

Dental Materia Medica and Therapeutics. By Herman Prinz, M.D., D.D.S., Professor of Materia Medical Therapeutics and Pathology, Washington University Dental School, St. Louis, Mo. A text book of nearly 600 pages for students and practitioners. Published by The C. V. Mosby Medical Book Publishing Co. of St. Louis.

This is probably the most pretentious book on this important subject of dental practice that has ever appeared. Dr. Prinz by his long training and experience as a worker in pharmacy and a teacher of dental students is perhaps as well qualified to do this work as any dental practitioner. The peculiar characteristic of the book is that, while it follows the classification as nearly as is possible of Wood's Therapeutics, it is an individual work, and hence may not meet the ideals of many students of this art. This individuality however gives a value to the work and lends an interest which fascinates the reader even when he can not always agree with the author's presentation. We do not mean by this that the author has not used other than his own material. He has in fact drawn generously on the work of all the well known producers of this as well as foreign coutries. He is a close reader of the German work especially and has taken many valuable things from these authors for this work. Dr. Prinz, however, has the faculty of using this material in connection with his own ideas in such a way as to give it an individual setting that is far more effecttive and forceful than the usual quoted matter. He has therefore made a book that will have a larger interest for the practitioner than most works on this subject will have. At the same time his systematic classification will prove a valuable help to the instructor in holding the interest of his students. The pharmacological method is largely followed in the treatment of all remedies, rather than the pharmaceutical, which makes so many of our text books on this subject such dry reading.

The first part of the work is devoted to a historical statement of the therapeutic art, its aim, and the use of drugs; classification, selection and administration of remedies; the pharmacy, prescribing and dispensing of drug remedies. All of which is very interesting and well done.

The second part is devoted to what the author terms Pharamo-Therapeutics, which means the authors comment on each drug is in proper place in the classification as to its pharmacolog and applied therapeutics. The author has succeeded admirably in adapting the scientific methods of Brunton, Cushney, Heinz and others to the treatment of dental pharmacology and he has from a wide experience and reading incorporated the best in our dental therapy.

The treatment of dental antiseptics is very comprehensively and scientifically presented. The classification is scientific and because of the authors skill made very practical, a feat, which few writers seem able to accomplish. If we were making this particular chapter we should be inclined to cut loose from the scientific formula and individualize still more than Dr. Prinz has seen fit to do. The whole subject of dental disinfection and sterilization in dentistry is markedly peculiar and needs its own statement to make it usable or comprehensive by the student or busy practitioner. We haven't space to make a detailed exposition of the treatment of all the several classes of remedies and can only say that no drug or approved remedy seems to have been omitted and all are given a sufficient and characteristic treatment that makes every page of the book worth reading. Because of his special interest in some remedies he offers more elaborate comment than on others. His treatment of anesthesia, both local and general, is most complete. One may feel that in general anesthetics nitrous oxid and ethyl chloride, while perhaps the generally used anesthetics in dentistry, do not give one as complete a statement of this class of anesthetics as our varied demands require. There are many cases of anesthesia for dental purposes which can not be best met with either Nitrous Oxide or Ethyle Chloride. For operation on cleft palates and the extraction of impacted third molars, Ether or Chloroform are preferable because of the time consumed in these operations.

In local anesthesia the author exhausts the subject from its drug as well as its technical aspect and leaves little more to be said.

The subject of Organ and Serum Therapy is not omitted, and while this subject at present has little definite in the way of well defined practice, the application of the principle in the treatment of peridental affections is being agitated and some statement should be welcomed in an up-to-date text book.

The important subject of physical therapeutics is given proper attention. This is an old treatment, but at no time has so intelligent use been made of physical remedies in the treatment of dental diseases as now.

There are of course as in all first editions, errors, typographical and rhetorical, which will be eliminated in time, there are also statements which will probably undergo modification, but it is a most valuable contribution to the subject, and the author and publishers deserve out best compliments for the excellent work they have done.

